# Numerical Modelling Of The V-J Combinations Of The T Cell Receptor TRA/TRD Locus

**DOI:** 10.1371/journal.pcbi.1000682

**Published:** 2010-02-19

**Authors:** Florence Thuderoz, Maria-Ana Simonet, Olivier Hansen, Nicolas Pasqual, Aurélie Dariz, Thierry Pascal Baum, Vivien Hierle, Jacques Demongeot, Patrice Noël Marche, Evelyne Jouvin-Marche

**Affiliations:** 1 CNRS, Laboratoire TIMC-IMAG, UMR 5525, Grenoble, France; 2 INSERM, Institut Albert Bonniot, Grenoble, France; 3 Université Joseph Fourier-Grenoble I, Faculté de Médecine, Grenoble, France; Utrecht University, Netherlands

## Abstract

T-Cell antigen Receptor (TR) repertoire is generated through rearrangements of V and J genes encoding α and β chains. The quantification and frequency for every V-J combination during ontogeny and development of the immune system remain to be precisely established. We have addressed this issue by building a model able to account for Vα-Jα gene rearrangements during thymus development of mice. So we developed a numerical model on the whole TRA/TRD locus, based on experimental data, to estimate how Vα and Jα genes become accessible to rearrangements. The progressive opening of the locus to V-J gene recombinations is modeled through windows of accessibility of different sizes and with different speeds of progression. Furthermore, the possibility of successive secondary V-J rearrangements was included in the modelling. The model points out some unbalanced V-J associations resulting from a preferential access to gene rearrangements and from a non-uniform partition of the accessibility of the J genes, depending on their location in the locus. The model shows that 3 to 4 successive rearrangements are sufficient to explain the use of all the V and J genes of the locus. Finally, the model provides information on both the kinetics of rearrangements and frequencies of each V-J associations. The model accounts for the essential features of the observed rearrangements on the TRA/TRD locus and may provide a reference for the repertoire of the V-J combinatorial diversity.

## Author Summary

Lymphocytes of the immune system ensure the body defense by the expression of receptors which are specific of targets, termed antigens. Each lymphocyte, deriving from the same original clone, expresses the same unique receptor. To achieve the production of receptors covering the wide variety of antigens, lymphocytes use a specialized genetic mechanism consisting of gene rearrangements. For instance, the genes encoding the receptor of the alpha chain of the T lymphocyte receptor (TRA) spread over a 1500 Kb genetic region which includes around 100 V genes, 60 J genes, and a single C gene. To constitute a functional alpha chain, one of the V and one of the J genes rearrange together to form a single exon. The precise definition of these V-J combinations is essential to understand the repertoire of TRA. We have developed a numerical model simulating all of the V-J combinations of TRA, fitting the available experimental observations obtained from the analysis of TRA in T lymphocytes of the thymus and the blood. Our model gives new insights on the rules controlling the use of V and J genes in providing a dynamic estimation of the total V-J combinations.

## Introduction

Functional antigen receptors expressed by T lymphocytes (TR) are generated during ontogeny by somatic recombination of gene segments coding for the variable (V), the joining (J), and the constant (C) segments [1]. The recombination mechanism is largely dependent on both the accessibility of the loci and the RAG enzymatic complex [2]–[5]. The murine TRA/TRD locus is composite, encoding TR α and δ chains and encompassed of more than 100 functional V genes [6]. In theory, each of the V genes may target one of the 49 functional J genes. The use of V and J genes during the process of recombination has been widely debated, and the studies support the consensus that V-J combinations are not random, with a use of J segments starting at the 5′ end (proximal to the V segments) and proceeding to the 3′ end [7]–[13]. The accessibility of the J region is controlled by the TR α enhancer (Eα), located at the 3′ end of the C gene [14] and by two promoters: i) T early α (TEA), located at the 5′ end of the Jα region and ii) J49 located 15 Kb downstream of TEA. Both of the promoters are activated by Eα [4],[5],[15]. Eα controls all the V to J associations whereas the two promoters are required for the rearrangements of the J genes situated at the 5′ end of the Jα region. However, the analyses of TEA-deleted alleles and those of blockade of TEA transcription showed significant alterations in J use and support the hypothesis that the TEA promoter can regulate both positively the promoters located in the first 12 Kb of J genes and negatively the downstream promoters [4], [15]–[17].

A particularity of the TRA locus is an absence of allelic exclusion [18] and its ability to undergo multiple cycles of secondary rearrangements [19],[20]. The process of successive rearrangements is stopped by either positive selection, which downregulates recombinase expression [21] or by cell death. Therefore, the impact of secondary rearrangements on the TRα gene assembly regulation remains to be defined.

Regarding the V and J gene use, it is suggested that the first V-J association targets the secondary one into a set of J segments located near the J segment involved in the primary rearrangement [5],[16]. The rules governing the use of the V genes have not been clearly elucidated. Nevertheless, observations converge to a consensus: the use of V segments would progress from proximal V genes, located near the J region, towards the V genes located in the distal region [9],[10]. At this point in time, the mechanism involved in the control of accessibility of V genes remains to debate [19].

The current state of the technology permits the analysis of some V-J combinations, essentially those at the extremities of the locus but still fails to establish a complete estimation of the V-J combinations. The main obstacle comes from the fact that some V genes are duplicated in similar copies in the V region central part, making problematic their unambiguous identification by molecular methods [22].

Consequently, numerical modelling of the V-J recombination process may offer valuable support to overcome the difficulty for accessing to a global view of TRA repertoire. For instance, if the J genes are chosen in a sequential way in the model, their use results unimodal, whereas it is known from experimental data that TRA/TRD locus displays two Hot Spots of recombination [2]–[5]. This discrepancy led us to build a mathematical model, parameterized from experimental data, on all V and J genes, including those in distal, proximal, and central positions. Confrontation between the data obtained from experiments and from modelling makes possible an estimation of dynamical parameters, such as the accessibility to rearrangements and the frequencies of the V-J associations, giving a more accurate estimation of the TRA combinatorial diversity.

## Results

The goal of building a model representative of the Vα-Jα associations was to reproduce the global biological features of T lymphocyte VαJα rearrangements occurring in the TRA/TRD locus with a software algorithm. This algorithm must be parameterized to find the conditions that reproduce the experimental data. Adequacy between biological and simulated results tells that all the essential aspects of the studied process were included in the model. This modelling approach led us to gather and search for some biological data about the parameters controlling the Vα-Jα rearrangement process in the mouse TRA/TRD locus.

### Update of the parameters controlling the mouse TRA/TRD locus utilization during rearrangements by an experimental approach

In order to build the model, we firstly required information about the physical position of the V and J genes in the TRA/TRD locus. These data were provided by IMGT (ImMunoGeneTics database; http://imgt.cines.fr/) and summarized in Table 1. In addition, we needed to define parameters such as the opening location (the position where the opening mechanism begins), the opening speed for the access to V and J genes and the opening duration. Two more parameters were added, a first maturation step in order to eliminate the TRD genes and an opening offset as we supposed a certain rigidity of the DNA chain, thus two genes placed very close from each other cannot rearrange together.

**Table 1:** TRA/TRD locus characterization.

	V region	J region
Length (Kb)	1300	64
Number of elements	104	60
Number of functional elements	100	49

#### Determination of the opening speeds for the V and J regions in the thymus

The data given in the first column of the Table 2 were obtained from rearrangements at the genomic level in BALB/c mice during thymic ontogeny and resume results presented in a previous work [9]. These data gave us the ontogeny days where V and J genes were first seen rearranged. In conjunction with physical gene positions, we calculated opening speeds that describe the progression of the accessibility to rearrangements over the Vα and Jα regions. Concerning the Jα region opening speed, in Fetal Fay 18th (F18), rearrangements of V19 with J61 to J48 corresponded to an opening of the J locus of 14773 bp in 24 h, thus the opening speed associated to these 24 h period is estimated to 615 bp/h. The same analysis was applied on F19, F20, and D0 (Day of birth). These data, fully presented in Table 2, showed that during ontogeny the opening speed of the J locus varies slightly, between 375 bp/h and 1150 bp/h, corresponding to an average opening speed of about 713 bp/h±3×396 bp/h with a 99.9% confidence interval.

**Table 2:** J locus accessibility: J genes seen rearranged to V19 during ontogeny.

Gestation day §	J opening	Opening distance #	Maximal opening Speed
F18 to F19	J61 to J48	14773 bp	615 bp/h
F19 to F20	J47 to J20	27618 bp	1150 bp/h
F20 to D0	J19 to J9	8998 bp	375–750 bp/h *
D0	J8 to J2	7940 bp	333 bp/h

**§:** Thymus from Fetal Day 18 (F18) to Day of birth (D0); data are analyzed from Figure 5 in Pasqual et al. [9].

# Length of the DNA sequence corresponding to the J opening.

*: For F20, the opening speed has been estimated between 375 bp/h to 750 pb/h depending on the offset of maxima 12 hours (9000 bp/24 h or 9000 bp/12 h).

For determining the Vα region opening speed, there were used single member V families at each V region extremity of the TRA/TRD locus. For instance, V1 and V2, the most distal from the J region, as well as V19, V20, and V21, located at the nearest extremity to the J region, were analyzed. We found rearranged proximal V genes from F18, while rearrangements of distal V1 and V2 genes (1300 Kb distant from the proximal V genes) were only detected from D0. Hence, the entire V region takes about 3 days to get wide opened, allowing us to estimate the overall “opening V-speed” as broadly 18 Kb/h (1300 Kb/(3×24 h)) with a 99.9% confidence interval of about 18±3×5 Kb/h.

#### V-J rearrangements in peripheral T lymphocytes

**Figure 1:**
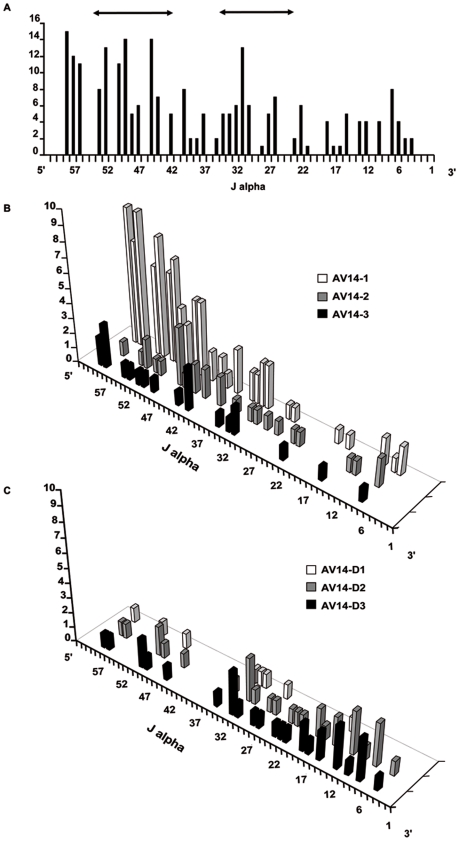
Quantification of the J region use by the V14 family. 254 V14 rearrangements were cloned from T lymphocytes, the V14 members and J genes were determined by sequencing [10]. (A) Profile of the J use by the six members of the V14 family. The two arrows indicate the localization of the two Hot Spots. (B) Profile of the three members the nearest from J genes and (C) J use by the most 5′ V14 members.

In order to complete the study of thymus repertoire, we extended the analysis of V-J rearrangements in peripheral T lymphocytes from spleen and lymph nodes of adult mice. We reported the J use of 254 sequences extracted from peripheral T lymphocytes that express V14 on their surfaces [10]. The V14 family is composed of 6 members spread from 1145 Kb to 492 Kb in the V region. The Figure 1 A shows the J use by the whole V14 family. This distribution is in accordance with the two Hot Spots reported by Rytkonen *et al.* [3],[23], which are indicated by two arrows over the histogram. Indeed, according to Rytkonen *et al.*, the J use distribution presents a preference for the J genes located over two regions named Hot Spots. The first Hot Spot (HSI) is located between J59 and J48 which corresponds to the region controlled by TEA; the second Hot Spot (HSII) is situated between J31 and J22. In addition, the Figure 1 presents the profiles of J used by the proximal V genes (V14-1, V14-2, and V14-3 on Fig. 1 B) and by the distal V genes (V14D-1, V14D-2, and V14D-3 on Fig. 1 C). These two histograms show that the proximal V to proximal J associations appear more frequent than the distal to distal associations and that the two Hot Spots are observed. Regarding the J region use, HSI is well observed for proximal V genes, and the HSII is well observed for distal V genes [10],[24]. After presenting these biological data, the results generated by the model will be exposed.

### Computational modelling approach

#### Values of the Parameters

Simulations were performed using two opening speeds chosen within intervals closed to the experimental 99.9% confidence intervals. For V speed, SV ∈ [0.35 Kb/h, 34 Kb/h] and for J speed, SJ ∈ [0.4 Kb/h, 1.55 Kb/h] with a mean opening speed of about 18 Kb/h for the V region and 1 Kb/h for the J region. The opening location of the simulation was fixed between the V and J genes in order to access directly to the TRAV and TRAJ genes after the first maturation, which was set to allow the elimination of the genes coding for TRD genes (region encompassing TRDV1 to TRDV5). Issues obtained from the modelling which best fit experimental data indicated that firstly, the duration of the first maturation step has a mean value of 5 hours, secondly, the number of successive rearrangements is 3 or 4, and thirdly, the opening duration before each rearrangement is 24 hours. The entire duration for the process of successive rearrangements is then 72 h or 96 h, which is in accordance with our data from ontogeny analyses (Table 2). When rearrangements were simulated by pairs, in order to account for the synchronization between the two alleles of individual cells, identical results were obtained.

#### Validation of the model by comparing simulated with thymic experimental data

**Figure 2:**
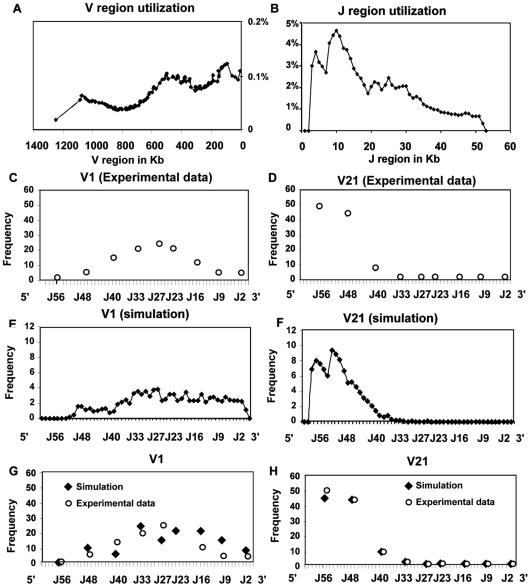
Validation of the modelling approach: analysis of the V and J region uses. (A) V region utilization: the X axis represents the V region in Kb. The Y axis shows the V gene percentage utilization in simulation. The simulated data sets have been normalized in order to be compared according to the following formula 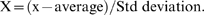 The fixed parameters of the simulation were as follow, one million of alpha chains, ongoing 1 to 4 rearrangements with opening speeds of 18 Kb/h and 1.03 Kb/h for the V and the J region respectively; (B) J region utilization: the X axis represents the J region in Kb; (C) and (D) Amplitude of J region utilization by opposite V genes, V1 (distal) and V21 (proximal). The X axis represents experimental quantification on 9 J genes. The Y axis shows the experimental utilization frequency of 9 J genes by the V1 and V21 genes. (E) and (F) Amplitude of J region utilization in the model. The X axis represents the J genes. The Y axis shows the model frequency utilization by each J genes. (G) and (H) Superposition of experimental and simulated data for the 9 J genes. The X axis represents experimental quantification on 9 J genes. V and J regions utilization from simulated data are similar to experimental data obtained from [9].

The results of the model simulations and its comparison with the thymic experimental data are presented on Figure 2. Firstly, we present the global V and J uses in the simulated population (Fig. 2, A and B). The simulation program provides frequencies of every V to J genes associations in a matrix form. The columns display the J genes and the rows the V genes. Every intersection column/row indicates the frequency of the considered V to J association. It is then possible to sum all the V-J frequencies where a single V gene is implicated by adding the corresponding entire row. By doing this with all of the V genes, the global V region utilization is calculated (Fig. 2 A), and the sums of every matrix columns result in the global J gene utilization (Fig. 2 B). Overall, the uses of the V genes decrease from proximal to distal V genes, and the J region uses decrease from proximal to distal J genes. These tendencies were experimentally observed from thymic [9] and peripheral data as well [7],[13].

Afterwards, we proceeded to quantify V1 and V21 rearrangements with a set of 9 J genes scattered along the J region in the thymus and compared these data with simulated outputs. Both experimental and modelling data show firstly that V1, which is the most distal V, has a low utilization rate of the proximal J genes and rearranges essentially the middle and distal J genes (Fig. 2, C, E, and G) and secondly that V21, which is the most proximal V, is mainly rearranged with the proximal J genes (Fig. 2, D, F, and H), following a Poisson distribution. The global utilization of J by V1 is similar in both experimental and simulated data. The frequencies from modelling are in correlation with the experimental nine J gene use (taken as representatives of the J region) by V1 and V21. In conclusion, the correspondence between data obtained by experimental analyses of rearrangements and those generated by *in silico* rearrangements validates the simulation program as model.

#### Program interface and generated graphics

**Figure 3:**
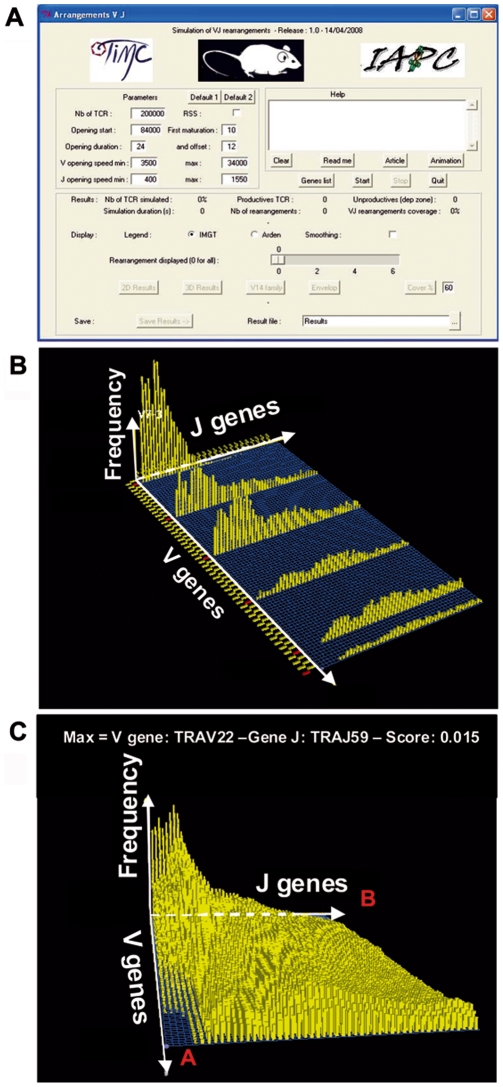
Model interface and results. (A) The main user interface window of the simulation program, (B) 2D representation of the rearrangement frequencies, (C) 3D representation of the rearrangement frequencies over all V and J gene associations.

**Figure 4:**
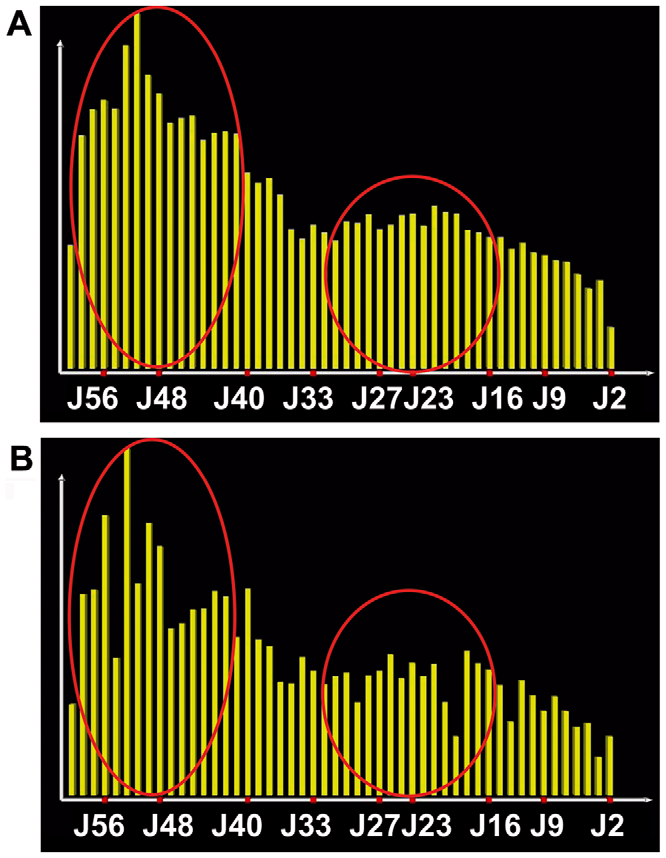
Representation of the V14 family rearrangement frequencies. Y axis represents the cumulated frequencies of all V14 genes with the J genes presented on the X axis, (A) without correction for RSS scores, (B) with correction accordingly to RSS scores. The two red ellipses show the localization of the two Hot Spots of recombination.

The simulation program with its user interface (Fig. 3 A) provides a 2-dimensional diagram showing a conditional V-J rearrangement distribution for different V from proximal, central and distal positions (Fig. 3 B). Moreover, a 3-dimensional histogram of V-J rearrangements representing the all TRα chain combinational repertoire can be generated (Fig. 3 C). The program offers as well an interesting graphic representation designed to plot the J region use by complex V families. For the V14 family, for example, the program displays the complete J use by all the 6 V members (Fig. 4 A).

#### Stimulation model for peripheral T cell repertoire

**Figure 5:**
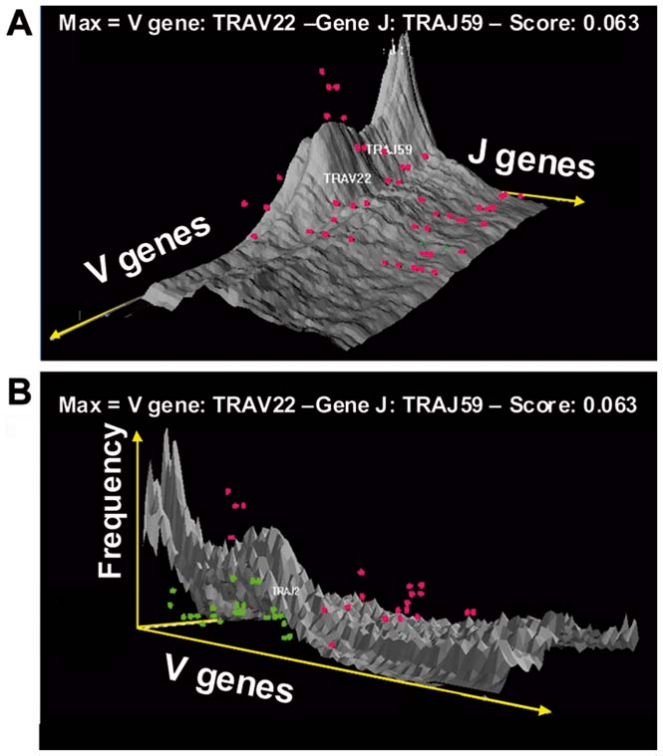
3-D superposition of V14 family rearrangements. (A) The fitted simulated data and (B) non fitted simulated data are shown in grey shapes. The experimental points above the simulation shape are represented in red. The experimental points under the simulation shape are represented in green.

In order to verify if the simulation model can be applied as a tool to determine the use of the different J by given V, we further quantified the use of 9 J by the 6 members of the V14 family in peripheral T lymphocytes. The resulting 54 V-J combinations, well spread along both the V and J regions, were plotted over the global V-J association 3-D graphic given by the simulated results (Fig. 5). For more precision, we plotted these frequencies of experimentally determined rearrangements over a fitted by 3-cubic spline fitting (Fig. 5 A) and a not fitted (Fig. 5 B) surfaces that interpolate the simulated frequencies. The experimental points over the surface appear in red, the ones under the surface appear in green. Beyond this visual adequacy, we demonstrated the accordance between simulated and experimental data by a numerical approach. We distinguished proximal, central and distal parts on the J region. For each of these parts, we compared the percentage of V14 rearrangements from experimental versus simulated data (Table 3). The V14 combinations with J61 to J48 represent 37% of the experimental data, and 35% of the simulated data, those with J47 to J24 stands for 46% and 50%, and combinations with J23 to J1 represent 17% and 15% of experimental and simulated data respectively. Noticeably, when the sums of simulated rearrangements are extended to all V, the uses of the J localized in the proximal, central and distal parts of the locus are in the same range than those found with V14 genes (last column of the Table 3). Additionally, the J use by the whole V14 family (Fig. 4 A) from our simulated repertoire presents a distribution where two Hot Spots are clearly visible. These Hot Spots are in accordance with our own observation from peripheral T-lymphocytes (Fig. 1 A) and the experimental data of Rytkonen *et al* [3],[23].

**Table 3:** J region use by V14: comparison between experimental and simulation data.

	Experiment	Simulation	
	V14 *	V14 #	All V #
J61 to J48	37%	35%	33%
J47 to J24	46%	50%	51%
J23 to J1	17%	15%	16%

*: Frequencies of rearrangements of V14 genes were calculated from Figure 2 in Aude-Garcia *et al.* [10], for the combinations with three J panels, corresponding to series of J genes scattered along the J region.

# Frequencies of rearrangements of V14 genes and of all V genes were calculated from modelling data for the combinations with same series of J genes.

#### New insights in the VJ repertoire given by the model simulations

Based on our modelling study, four features concerning the V-J rearrangement process emerge. First of all, concerning the frequencies of the associations, the main information supplied by the model is that 96% (4704 out of 4900) of the V-J associations are probable. Hence, two areas where V-J combinations rarely occur could be defined (Fig. 3 C): the “A” area as associations between proximal V and distal J and the “B” area as associations between distal V and proximal J. These occasional associations are a consequence of a non-synchronized availability for rearrangements of the concerned V and J genes, as already documented in experimental data [7],[13],[19]. Secondly, the model gives estimation for the frequencies of each V-J combination building the whole combinatorial repertoire shape. In the third place, the model states the influence of the Recombination Signal Sequence (RSS) on the V-J association distribution, which remains until now debated [25],[26]. For that purpose, we ran simulations with and without taking into account the RSS scores (available in IMGT). The 2D graphs of the V14 family repertoire (Fig. 4, A and B) show that introducing variations accordingly to the RSS scores (Fig. 4 B) does not drastically affect the shape of the global repertoire distribution but leads to a local effect on certain J genes. The algorithm for choosing the J genes regarding their RSS score values has been favorably tested by using the Monte Carlo method. In the fourth place, the model provides information on the contribution of each wave of successive rearrangements to account for the total of V-J associations. Therefore, we tested the occurrence of 1 to 6 successive rearrangements in simulations. With 1 or 2 rearrangements, only the proximal V to proximal J associations are observed. With 5 or more rearrangements, the repertoire presents a distal border effect, corresponding to numerous rearrangements of the distal V and distal J regions, which are incoherent with experimental data. In conclusion, the overall repertoire generated by the simulation is in accordance with experimental data only by allowing 3 to 4 rearrangements, with a delay of 24 hours per rearrangement. Moreover, the contribution of each wave of successive rearrangements appears to decrease accordingly to their rank; 40% of the overall V-J associations is produced by the first wave of rearrangements, 33%, 19% and 8% come from the subsequent rearrangement waves respectively (Table 4).

**Table 4:** Contribution of each rearrangement round into the total V-J combinatorial repertoire.

Rearrangement	First	Second	Third	Fourth
Percentage	40%	33%	19%	8%

#### Robustness of the model

**Figure 6:**
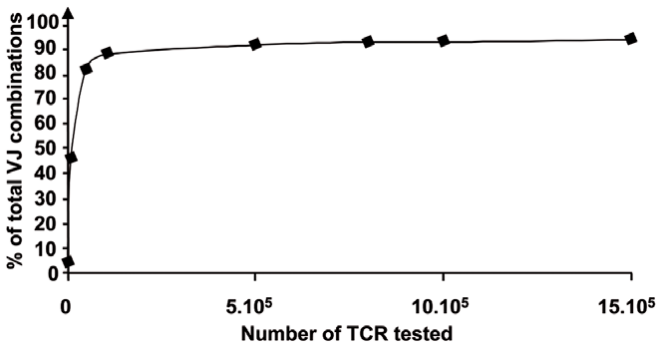
Combinational diversity of V-J combinations and population size. X axis represents the number of TR tested in the simulation, and Y axis indicates the percentage of the number of the different V-J combinations obtained by the simulation over the total number of possible V-J combinations.

To assure that the sampling size used in simulations was sufficient, we checked the representativeness of the repertoire by making sets of simulations ranging from 10^2^ to 1.5×10^6^ rearrangements. We found that diversity became relevant when the population size was higher than 5×10^5^ rearrangements. This showed the pertinence of a repertoire calculation based on a 10^6^ alpha chains population. A combinatorial diversity graph is plotted in Figure 6. Variations of about 5 to 10% in the values of the parameters (such as the intervals of the opening speeds Sv and Sj, opening duration, and offset) provided simulation results in concordance with the experimental data. However, larger variations in the values of the parameters induced major deviations (not shown) on the modelling simulation results compared to experimental data.

Finally, the consistency between simulated data and the frequencies observed in the thymus and the periphery validates our model as a relevant tool accounting for the mature repertoire of TRA/TRD.

## Discussion

**Figure 7:**
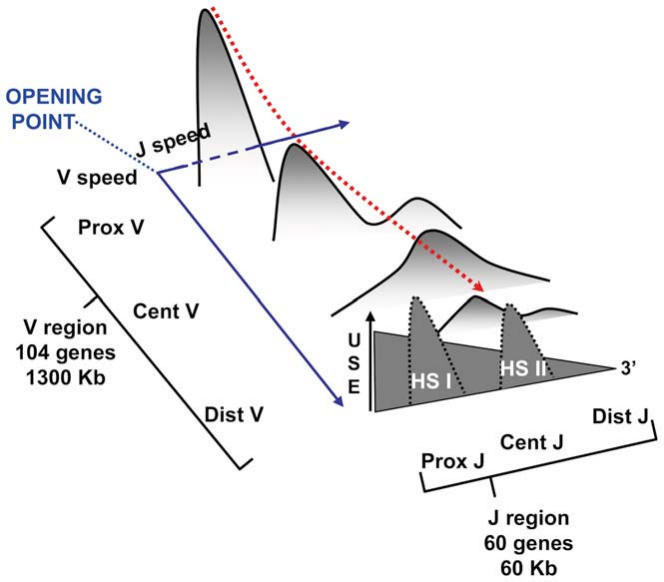
Schematic representation of the TRA/TRD locus use. The scheme shows the rearrangement distribution of 4 V genes with all J genes. The dashed red arrow indicates the decreasing frequency of rearrangements which correlates with the associations of distal V and J genes. A distal V gene is very rarely rearranged with proximal J genes because of the high recombination frequency between proximal V and J genes, leading to the proximal J genes deletion. 1) the first step governing the TRA/TRD locus utilization is defined by the opening of the most proximal V-J region, 2) According that T cells can undergo 1 to 3 secondary rearrangements, the second step giving the V and J accessible windows, is defined by the opening speeds of V and J regions. V speed is about 18 Kb/h whereas J speed is about 1 Kb/h, 3) J region has two Hot Spots of rearrangement. HSI is centred on J48 and HSII is centred on J30.

This article focuses on a new approach to account for the features of the V to J rearrangement process in the TRA/TRD locus as well as to give a first estimation of the combinational repertoire in a 3-dimension representation. To accomplish this purpose, we have defined a mathematical model fitting experimental observations obtained from T lymphocytes rearrangements in the thymus. Jointly, the experimental data and the mathematical model made possible the interpretation of the mouse T-cell alpha chain repertoire characteristics. The evolution of the shapes for the V-J rearrangement frequencies in the simulations, presented in Figure 7, showed a transient bi-modal shape corresponding to the Hot Spots of V-J recombinations as observed in our experimental data and literature [3],[10]. Furthermore, the model results also fit with V-J rearrangements obtained from T lymphocytes of the periphery. Therefore, our model provides a major improvement to previous attempts of simulation of the TRA combinatorial repertoire building [27].

### 

#### Opening velocities and gene density

Our model is based on the fact that V and J regions are used in a progressive and decreasing manner from 3′ to 5′ for the V region and from 5′ to 3′ for the J region. Our quantitative approach points out that the physical position of the genes is the main structural parameter governing the uses of V and J genes. The J and V regions become accessible from proximal to distal genes according to an average “opening speed” of approximately 1 Kb/h for the J region and around 18 Kb/h for the V region. Interestingly, this difference between the V and J region opening speed values can be related to the gene density. In fact, the number of genes and the size of the V and J regions differ significantly. As a matter of fact, the V region length is 20 times larger than the J one (∼1300 Kb versus ∼60 Kb), but the density of J genes is about 10 times higher than the density of the V genes (1 J by Kb versus 0.13 V by Kb). Consequently, the opening speeds calculated in terms of genes per hour is 1.4 for the V region and 0.83 for the J region (calculated as follows: the number of genes in the locus times the speed in Kb per hour, then divided by the length of the locus). Finally, taking into account the gene density of each region, the opening speeds of the V and J regions are almost identical. Our observations reinforce two putative scenarios being previously proposed to explain the opening of the J and V regions respectively. The first one, the J local service scenario was proposed for the J region [28]. It consists in a J use that follows small steps during the successive rearrangements. This local service is controlled by promoter activities associated to some of the other J genes. The second one, the V region express service proposes a large window for the gene accessibility to rearrangements controlled by enhancer activity. It reflects the larger utilization of a V region, whose genes are more scattered than those in the J one [9]. In addition, the speed calculations also take into consideration the regulatory elements controlling the gene accessibility [29]. As a remark, a parametric study with different speed values indicates that the proposed speeds are the only ones allowing a use of V and J regions correlating precisely with the experimental results.

#### Combinatorial repertoire distribution

Given that the totality of the V-J combination frequencies is computable in the framework of our model, we can visualize the whole simulated TRA combinatorial repertoire and thus estimate each V-J association frequency. Indeed, the probability of any combination is given by selecting a specific V-J combination on the 3D graph (Fig. 3 C). Table 5 displays the probability of nine V-J combinations selected along the locus. Regarding the positions of the V and J genes over the locus, the probability of association varies between 0.01 for any V to distal J and 0.198 for any V to proximal J, highlighting the fact that a V to proximal J combination is about 20 times more probable than a V to distal J association.

**Table 5:** V-J association probabilities along the TRA locus.

	V21 Proximal	V4-2 Central	V2 Distal
J52 Proximal	0.148	0.050	0
J31 Central	0.002	0.029	0.023
J2 distal	0	0.003	0.007

9 V-J association probabilities given by the model. These results show an unbalanced use of the proximal and distal V and J genes. For instance, if all V-J combinations are equiprobable, the probability of each V-J association should be about 2.10^−4^.

Moreover, this 3D graphic points out that one central area in the V-J association plane contains the most represented combinations of the repertoire (Fig. 3 C). Furthermore, two areas (A and B in Fig. 3 C) reveal rarely represented V-J combinations, due to the fact that the V and J genes involved in these combinations are not accessible to get rearranged simultaneously. Concerning the A area, when the proximal J genes are recombining, the distal V genes are still inaccessible, and when they become accessible, the proximal J genes are deleted because of previous rearrangements. Similarly, on the B area, the non-synchronized accessibility of the proximal V genes and the distal J genes explains that their associations are not observed in the simulation.

#### The J region use confirms the existence of two Hot Spots

**Figure 8:**
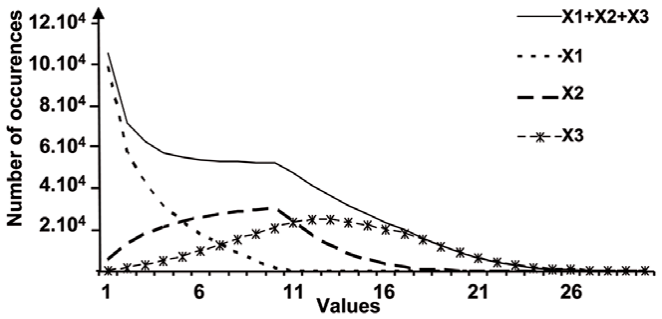
Successive rearrangements and building of the combinatorial repertoire shape. Three successive draws of random integers were done successively, the first one giving an integer x1 between 0 and 10 following a Poissonian law. The second and the third ones follow a Gaussian law, the second giving an integer x2 between x1 and x1+10, and the third giving an integer x3 between x2 and x2+10, and that 300 000 times. The first, second and third curves were added to build the sum curve.

**Figure 9:**
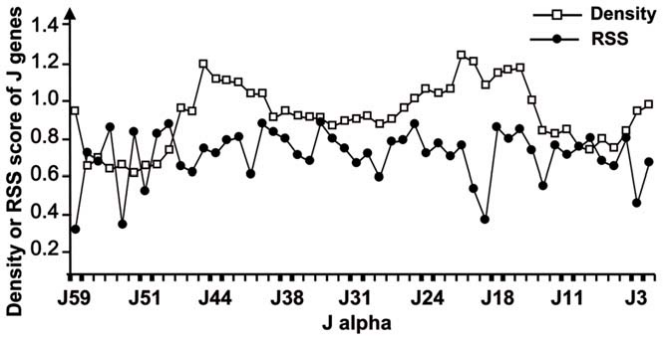
Density and RSS scores of the J genes. Values for the density (open squares) and RSS scores (dark circles) were calculated, as described in methods, for each J gene from the four previous and next genes. X axis represents the J genes, the Y axis the density or the RSS score for all J genes.

The progressive opening mechanism over the TRA locus provides V-J combinations that show a specific pattern; each given V gene is rearranged with a contiguous set of J genes. The distribution of these J genes presents a Poissonian distribution for the proximal V genes or a Gaussian shape for the distal V genes. The changes in the J use are progressive, depending on the V position: the more distal is a V gene, the more distal and larger is the set of J genes used, and the less represented are these V-J associations in the whole V-J repertoire (Fig. 3 C). There are two main probabilistic bases for the occurrence of the Hot Spots in the J region observed in the simulation results. The successive rearrangements are achieved by consecutive random choices of J genes, considering the progression of their access to recombination and optionally their RSS score values. The first J gene choice, corresponding to the first rearrangement, follows a Poissonian law whereas individually the two other J gene choices follow a Gaussian law (Fig. 8). Altogether, the consecutive random choices of J genes build a multimodal curve of occurrence, which allows the appearance of two Hot Spots (Fig. 8, solid line). The first Hot Spot results from the Poisson's distribution of the first J choice and the second one from the Gaussian distributions of the subsequent J choices. Moreover, it is important to remark that the density of J genes in the TRA locus is not uniform: J genes are less dense between J58-J47, J39-J28, and J14-J4, whereas they have a higher density between J45-J40 and J24-J15 (Fig. 9). The J gene density reaches its maximum in the area around J21-J22, corresponding to the place of the second Hot Spot. Interestingly, we observed the Hot Spots when the model parameter values were in the range where the opening dynamics allowed more frequent rearrangements within the areas of maximal gene density.

#### Number of secondary rearrangements

Secondary V-J rearrangements of the TRA/TRD locus are widely accepted [20],[21],[28],[30],[31]. However, the number of plausible secondary rearrangements remains unknown. Our model predicts that after the first maturation step of the TRA/TRD locus, consisting in the elimination of the TRD genes, the first V-J rearrangement of TRA is followed by a maximum of three secondary rearrangements. Nevertheless, each round of rearrangement contributes differently in the building of the whole repertoire, decreasing after each wave, and consequently, the fourth rearrangements have a weak contribution of 8%. This estimation of 4 total rearrangements is based on a realistic model including opening speeds from ontogenic experiments and may be more precise than Warmflash theoretical model's results that proposes a higher number of successive rearrangements [27]. It is important to consider that the number of secondary rearrangements can be affected by the lifespan of the rearranging T lymphocytes. For instance, the RORγ-deficient mice, presenting a shorter lifespan of DP thymocytes, show essentially proximal J genes rearranged, while Bcl-xL–transgenic mice, having DP with a longer lifespan, present a higher rate of distal J genes rearranged [28].

#### RSS influence

Regarding the Recombination Signal Sequence (RSS) influence, our model is able to incorporate the RSS diversity information through scores. The simulations using these RSS scores show a local quantitative influence but do not change the global profile of the frequency curves (Fig. 5, A and B). In conclusion, the RSSs may only influence local specificities within the accessibility windows moving across the TRA/TRD locus in a bi-directional way. This is in good accordance with mouse TRB locus observations showing that V gene RSSs neither correlate with any particular restriction of J genes nor with any high V-J rearrangement frequencies [32].

#### The sequential windowing model: a tool to determine the peripheral V-J association frequencies

We previously observed experimentally and tested statistically that the thymic and the peripheral repertoires showed similar profiles of J uses by the V14 family [10]. When we compared the experimental data of the uses of J genes by the V14 family members we found that the J use profiles fitted our model results. It is acknowledged that the V14 is a multimember family and may be representative of the J use by different V genes. Finally, our data suggest that the model would be used as a tool to determine the V-J association frequencies in the peripheral T lymphocytes.

All these remarks support the realistic character of our model, which includes the essential features of the V-J rearrangement process in the TRA/TRD locus. In conclusion, the combination of experimental and mathematical approaches gives new insights on combinatorial repertoire biases due to non-equiprobable V-J combinations in TRA/TRD rearrangements, and allows defining more accurately the TRA/TRD primary combinatorial repertoire. In the future, the model could be adapted to other loci and other species, to propose accurate estimations of the V-J combinatorial diversity, giving a dynamical vision of the immune diversity generation during differentiation of T cells and B lymphocytes.

## Materials and Methods

### 

#### Nomenclature

Official nomenclature for V and J genes is chosen according to the IMGT database (http://imgt.cines.fr). NCBI (National Center for Biotechnology Information) accession numbers are AE008683-AE008686 for the mouse V region and M64239 for the J region. Positions of each V and J genes were calculated based on these data as previously described [6].

#### Mouse

BALB/c mice were purchased from Charles-River (L'Abresles, France). Mice were housed and humanely killed according to relevant national guidelines. No experimental work was done on living animals. Fetal thymi were obtained from timed pregnancies, where Fetal Day 1 (F1) corresponds to the day of detection of a vaginal plug. Thymic lobes from embryonic mice were pooled and mechanically dissociated in PBS before DNA extraction.

#### Multiplex PCR assay analysis

multiplex PCR assays and quantification analysis were done as described in [9],[33],[34].

#### Quantitative PCR

Real time PCR were performed on a Light CyclerTM (Roche diagnostics, Meylan, France). The specificity of the unique amplification product was determined by melting curve analysis and using migrations on agarose gels followed by southern blotting with the corresponding internal V probes [9].

### Description of the computational model

**Figure 10:**
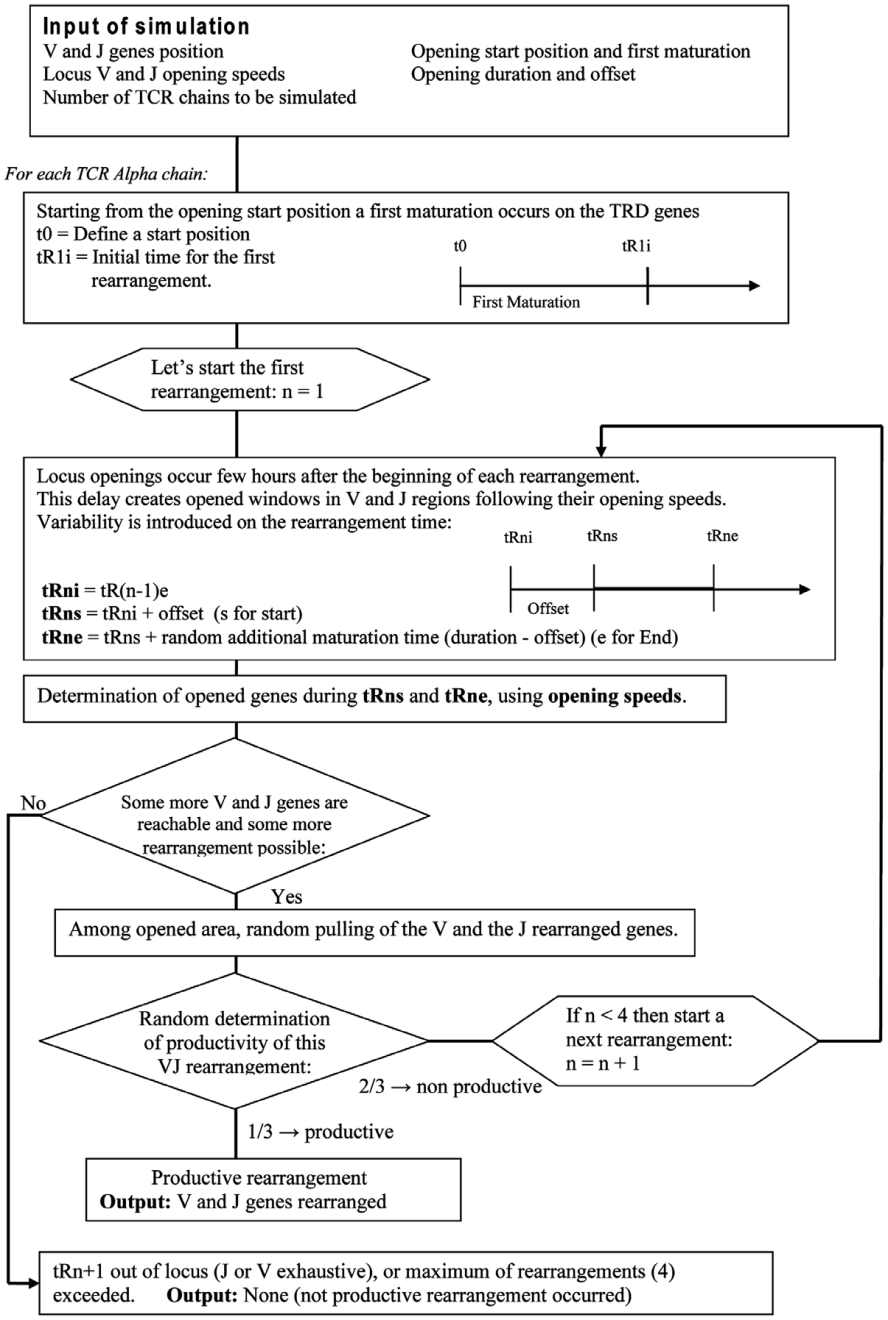
Flow diagram for the sequential windowing model algorithm.

Our **sequential windowing model** is a specific model of successive windows, each step corresponding to a differential motion of the window extremities, with opening velocity faster on the V side than on the J one. The simulation of the V-J rearrangements in the TRA/TRD locus is based on a computational occurrence discrete model using parameters determined from experimental data (Fig. 10). The model consists in dynamical rules depending on constant (structural) and experimental parameters. The constant parameters are the physical positions of the 100 V and 49 J functional genes and the first TRA/TRD locus opening points. The varying parameters (whose sensitivity will be studied by simulation) are the opening speed intervals of the V and J regions, the opening duration before each rearrangement, and the opening offset.

We define variables and dynamical rules of the model as follows:

1. **The physical positions of V and J genes in the TRA/TRD locus** are calculated by measuring the genomic distance from the TRAC chain.
2. **The first maturation step** is fixed before the first rearrangement allowing the deletion of the region encompassing TRDV1 to TRDV5 including the TRD locus. Its duration value is a constant parameter, which was determined by simulation varying values between 0 hours and 10 hours.
3. **The opening location** describes the site where the opening mechanism begins. This site is fixed at the TEA location [35].
4. **The opening speeds** of the V and J genes calculated above are denoted respectively by S_V_ and S_J_. They are random variables between a minimum and a maximum. Each TCRA locus is simulated independently to be consistent with the absence of allelic exclusion. Furthermore, rearrangements were also simulated by pairs using the same V and J opening speeds, in order to account for the synchronization between the two alleles of individual cells.
5. **The Opening duration before each rearrangement** is a constant parameter whose value was determined through simulations, by varying values between 2 hours and 50 hours.
6. N denotes the number of authorized **secondary rearrangements** during the simulation. It is a varying parameter as well, values between N = 0 to N = 6 were studied by simulations.
7. The **probability to perform an in-frame rearrangement** at any step k (1≤k≤N) is fixed to 1/3 that is the maximal possible value. If the rearrangement is randomly determined in-frame, the procedure is stopped for this locus and the V-J association generated is stored. If the simulation gives an out-of frame rearrangement at the step k, a new secondary rearrangement is randomly executed at the step k+1 on the available part of the locus. The new window of accessibility is calculated in base of the “**opening speeds**” and the **“opening duration before each rearrangement”** parameters. This successive rearrangement procedure remains until either an in-frame rearrangement occurs or k equals the maximum number of rearrangements N.
8. We refer to the length of the window of accessible DNA over the V region at a step k as LV_k_ (and LJ_k_ for the J window length), These windows progress from the proximal to distal extremities of the TRA/TRD locus V and J regions. The LVs and LJs verify the equations:
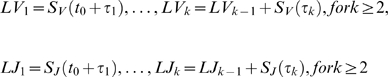
where the opening offset time t_0_ denotes a minimal time of opening and τ_k_ (k≥1) are random variables uniformly distributed between t_0_ and the end of the opening process.
9. For every rearrangement occurrence, we define for each gene V_i_ (J_j_ respectively) a Boolean variable BV_ik_ (resp. BJ_jk_) equal at the k^th^ rearrangement to 1 if the gene is open (“accessible”), and to 0 if it is closed (“non-accessible”) or if it has been deleted during a previous rearrangement of order i (i<k).
10. The RSS score KV_i_ (resp. KJ_j_) represents for each gene V_i_ (J_j_ respectively) the homology percentage compared to a consensus sequence. This score basically takes value p, 0≤p≤1, if there is p % of identity between the RSS and the consensus proposed by Glusman *et al*. [25], allowing us to estimate a RSS identity score ranging from 0.3 to 1, where 1 corresponds to a fully consensus RSS (see additional data). Our RSS score is in agreement with the status of functional versus pseudo V or J gene (0.3< pseudo rearrangement score <0.65; 0.65< functional score <1). According to its non-functional status, a J-pseudo recombination is never found rearranged and consequently the corresponding J-RSS score is assimilated to zero in simulations. RSS score is equal to 1 when simulation is done without taking account the RSS. We call FV_k_ (resp. FJ_k_) the distribution function (i.e., the relative length) obtained after the k^th^ rearrangement by adding the BV (resp. BJ) variables:
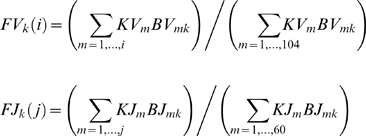

11. At step k, we choose the distribution functions FV_k_ and FJ_k_ corresponding to the random variables RV_k_ (resp. RJ_k_) uniform on [0,1] and we calculate a number NVk (resp.NJk) equal to inf(FV_k_^−1^(RV_k_)) (resp. inf(FJ_k_^−1^(RJ_k_))). NVk and NJk corresponding to the V and J genes to rearrange.

The **number of simulated TRAD loci** gives the size of the simulated population. In the figures shown in this paper, 1 million of V-J rearrangements have been simulated.

The **simulation Output** is presented in a matrix form incremented by the successive V-J in-frame rearrangements. Final results show the total number of V-J combinations available at the end of the whole simulation. These results can be plotted in different 3D representations using the interface. It is also possible to display the results for multi member V famillies corresponding to the real time PCR's.
